# Supplementary figures and images for: Changes in the urinary proteome before and after quadrivalent influenza vaccine and COVID-19 vaccination
Source: Front Immunol. 2022 Oct 7;13:946791. doi: 10.3389/fimmu.2022.946791 (PMC9585259; doi:10.3389/fimmu.2022.946791)

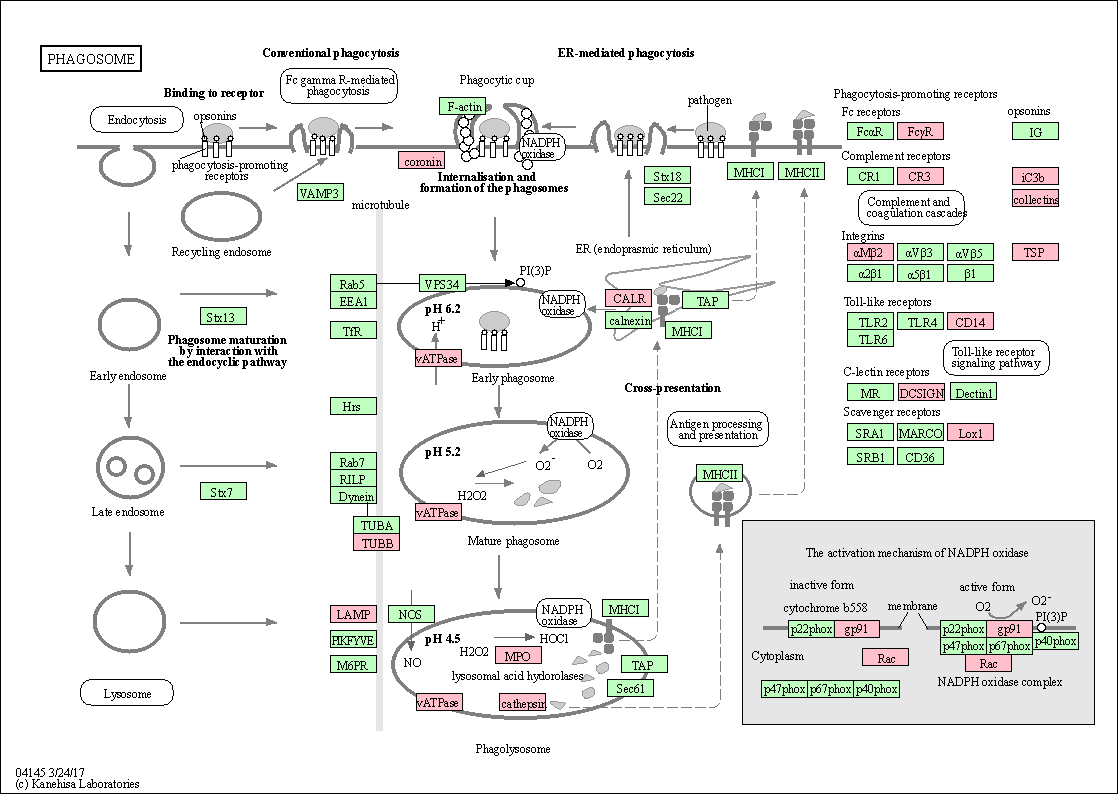

Supplement: Supplementary file 9 [file Image_1.png]

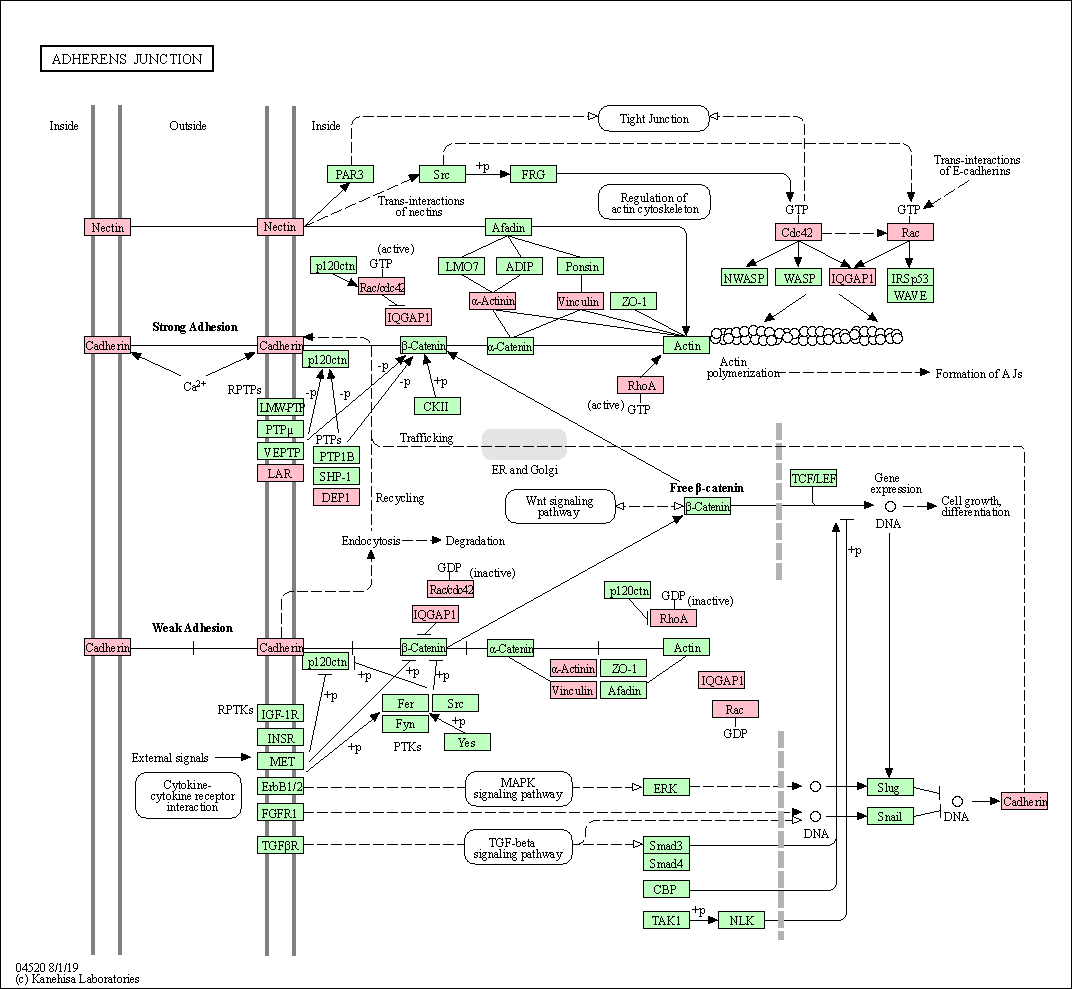

Supplement: Supplementary file 10 [file Image_2.png]

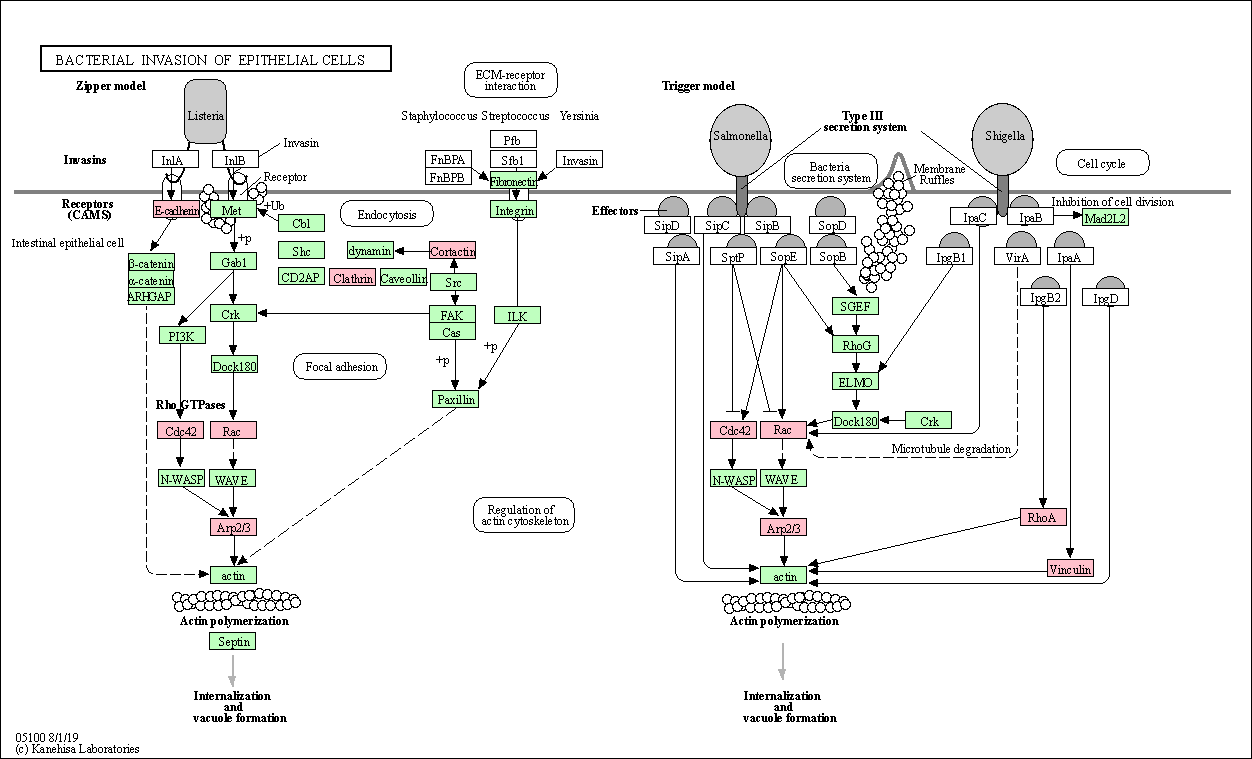

Supplement: Supplementary file 11 [file Image_3.png]

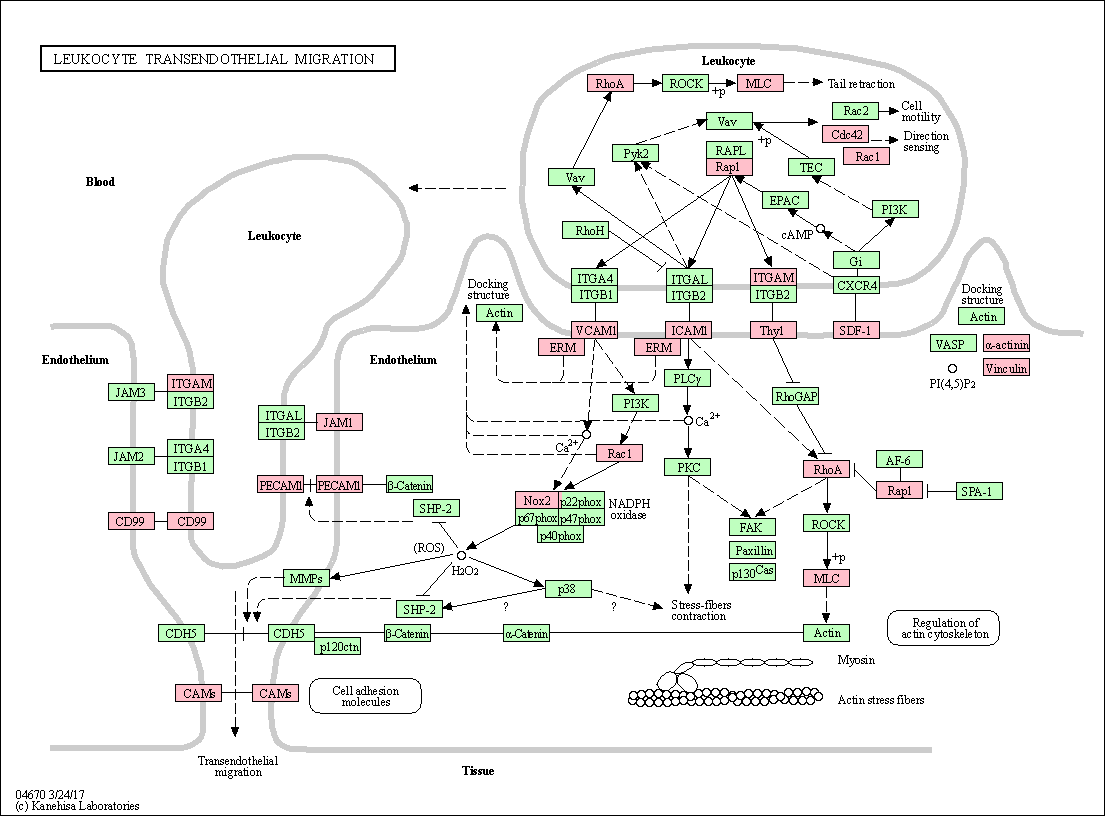

Supplement: Supplementary file 12 [file Image_4.png]

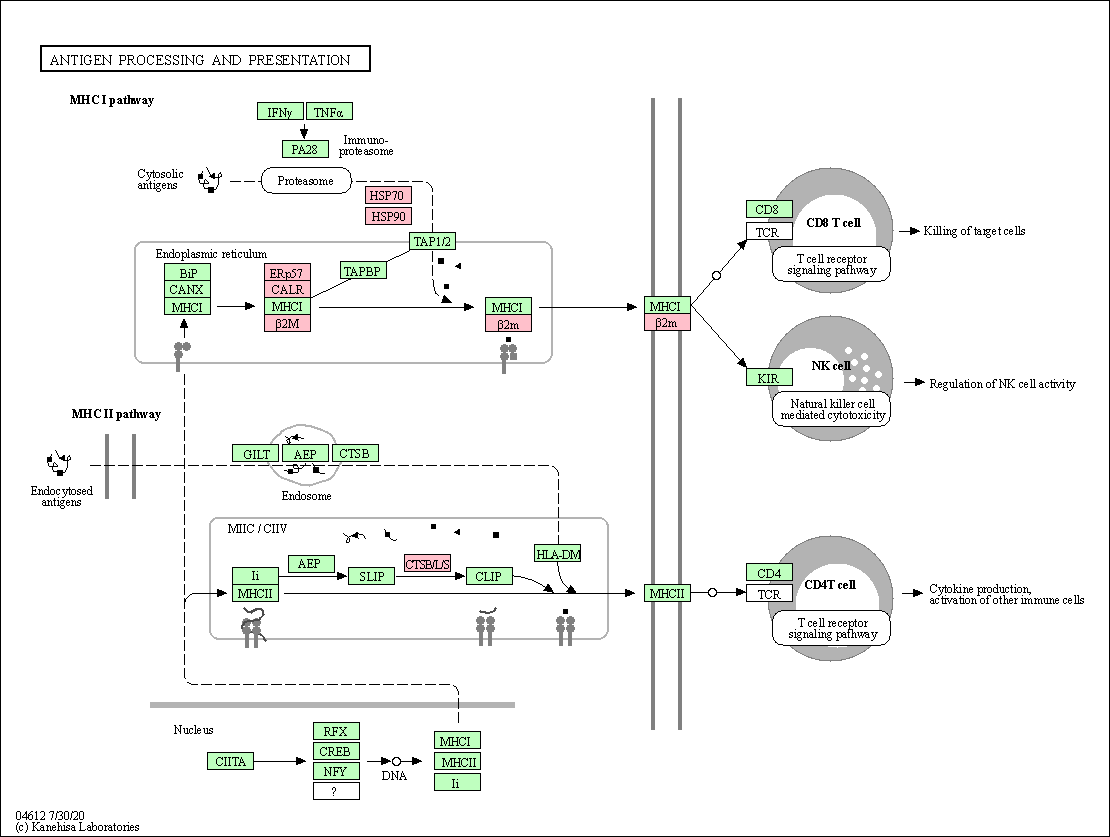

Supplement: Supplementary file 13 [file Image_5.png]

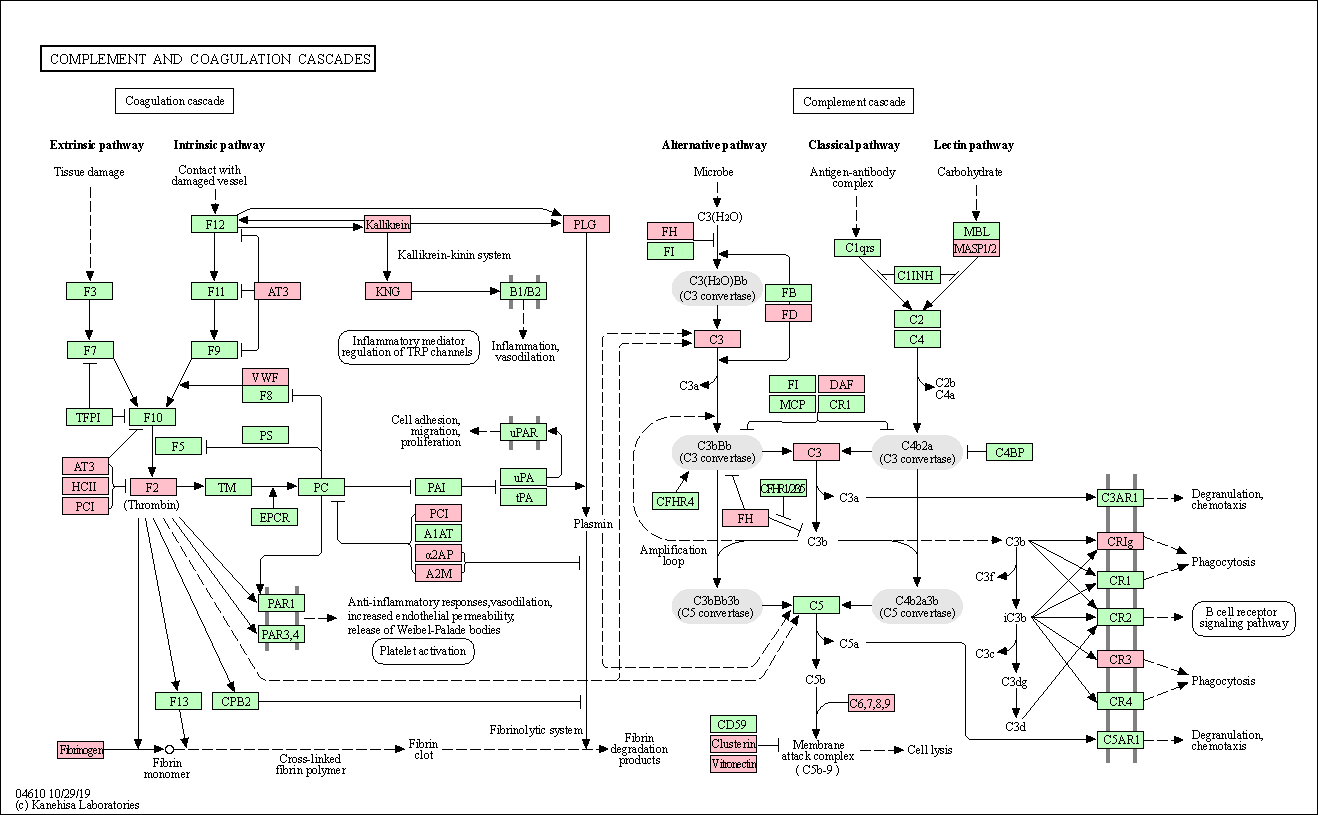

Supplement: Supplementary file 14 [file Image_6.png]

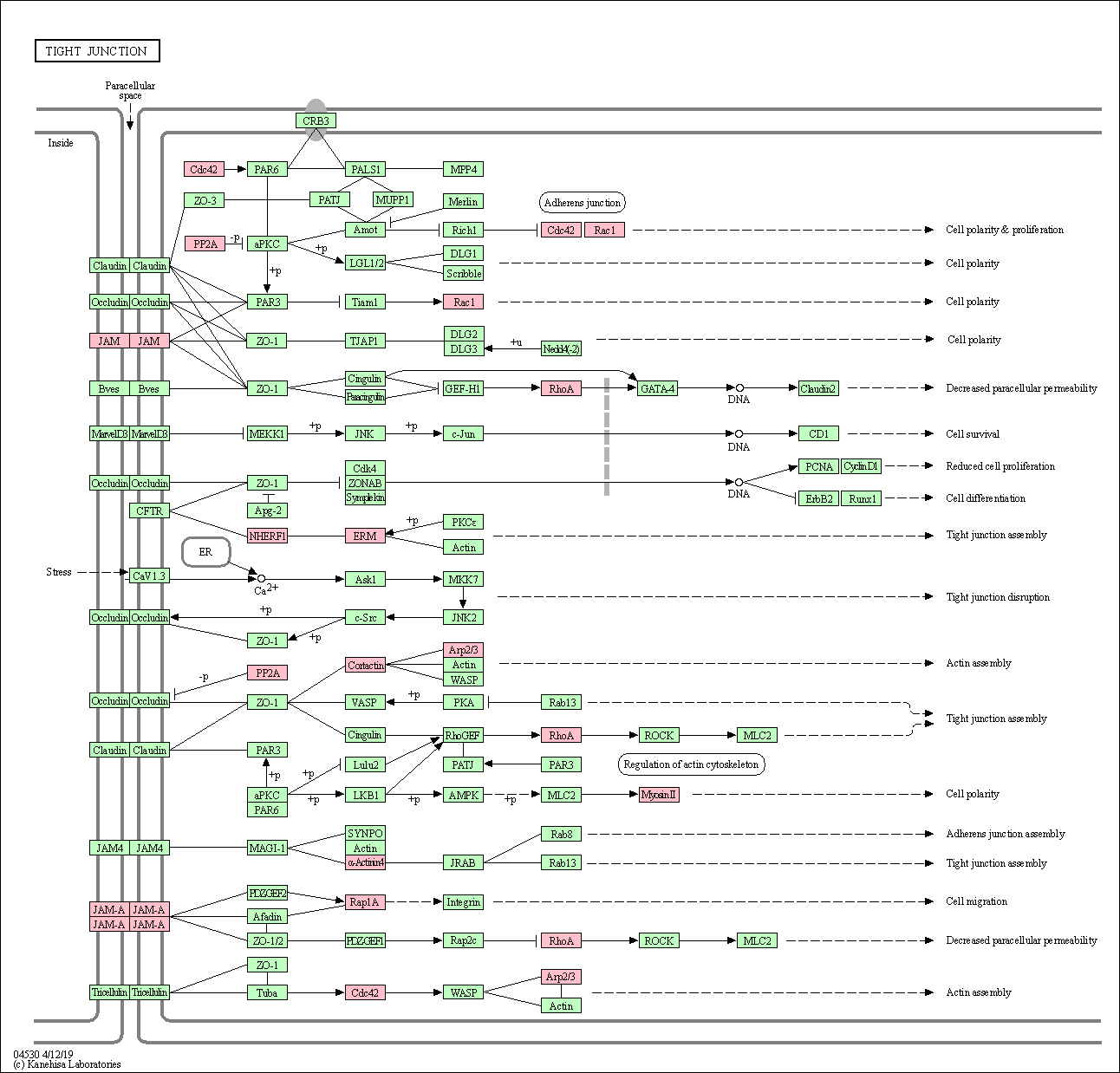

Supplement: Supplementary file 15 [file Image_7.png]

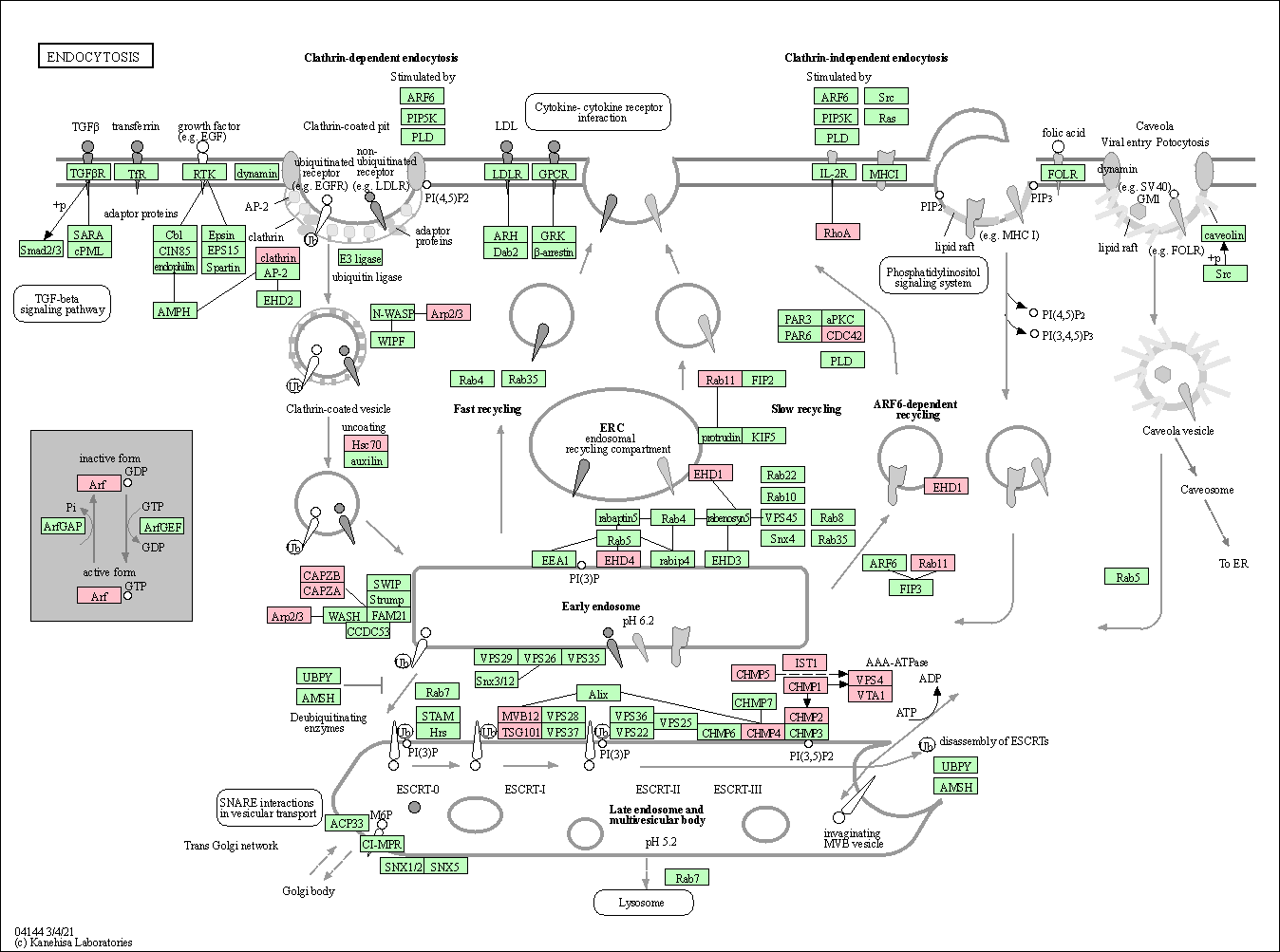

Supplement: Supplementary file 16 [file Image_8.png]
